# Comparison of DSM-5 and proposed ICD-11 criteria for PTSD with DSM-IV and ICD-10: changes in PTSD prevalence in military personnel

**DOI:** 10.1080/20008198.2017.1386988

**Published:** 2017-10-17

**Authors:** Annika Kuester, Kai Köhler, Thomas Ehring, Christine Knaevelsrud, Louisa Kober, Antje Krüger-Gottschalk, Ingo Schäfer, Julia Schellong, Ulrich Wesemann, Heinrich Rau

**Affiliations:** ^a^ Department of Clinical Psychology and Psychotherapy, Freie University Berlin, Berlin, Germany; ^b^ Psychotrauma Centre, German Armed Forces Hospital Berlin, Berlin, Germany; ^c^ Department of Psychology, Ludwig-Maximilians-University Munich, Munich, Germany; ^d^ Department of Psychological Assessment, Methodology and Legal Psychology, Friedrich-Alexander-University Erlangen-Nürnberg, Nürnberg, Germany; ^e^ Institute of Psychology, University of Münster, Münster, Germany; ^f^ Centre for Interdisciplinary Addiction Research, University of Hamburg, Hamburg, Germany; ^g^ Department of Psychiatry and Psychotherapy, University Medical Center Hamburg-Eppendorf, Hamburg, Germany; ^h^ Department of Psychotherapy and Psychosomatic Medicine, Technical University Dresden, Dresden, Germany

**Keywords:** Posttraumatic stress disorder, DSM, ICD, military, epidemiology, prevalence, concordance, PTSD, trastorno por estrés postraumático, DSM, CIE, Militar, Epidemiología, prevalencia, concordancia, TEPT, 创伤后应激障碍, DSM, ICD, 军队, 流行病学, 患病率, 一致, PTSD, • The consistency between provisional PTSD prevalence according to DSM-IV, DSM-5, ICD-10, and the proposed ICD-11 criteria was examined in a sample of 100 members of the German Armed Forces.• The provisional PTSD prevalence was the same under DSM-IV and DSM-5, and comparable under DSM-5 and the ICD-11 proposal, indicating a satisfactory agreement between these systems.• The provisional PTSD prevalence was significantly increased under the ICD-11 proposal compared to ICD-10, mainly due to the deletion of the time criterion in the ICD-11 proposal.• Preliminary support for the assumption of a concise set of six PTSD core symptoms and for the deletion of the time criterion as presented in the ICD-11 proposal.• Satisfactory consistency between preliminary PTSD prevalence based on DSM-IV, DSM-5, and the ICD-11 proposal and overall support for the changes made to DSM and ICD.• Future research needs to examine what diagnostic requirements are necessary and sufficient for diagnosing PTSD and whether these are approximated by the ICD-11 proposal.

## Abstract

**Background:** Recently, changes have been introduced to the diagnostic criteria for posttraumatic stress disorder (PTSD) according to the Diagnostic and Statistical Manual of Mental Disorders (DSM) and the International Classification of Diseases (ICD).

**Objectives:**This study investigated the effect of the diagnostic changes made from DSM-IV to DSM-5 and from ICD-10 to the proposed ICD-11. The concordance of provisional PTSD prevalence between the diagnostic criteria was examined in a convenience sample of 100 members of the German Armed Forces.

**Method:** Based on questionnaire measurements, provisional PTSD prevalence was assessed according to DSM-IV, DSM-5, ICD-10, and proposed ICD-11 criteria. Consistency of the diagnostic status across the diagnostic systems was statistically evaluated.

**Results:** Provisional PTSD prevalence was the same for DSM-IV and DSM-5 (both 56%) and comparable under DSM-5 versus ICD-11 proposal (48%). Agreement between DSM-IV and DSM-5, and between DSM-5 and the proposed ICD-11, was high (both *p *< .001). Provisional PTSD prevalence was significantly increased under ICD-11 proposal compared to ICD-10 (30%) which was mainly due to the deletion of the time criterion. Agreement between ICD-10 and the proposed ICD-11 was low (*p *= .014).

**Conclusion:** This study provides preliminary evidence for a satisfactory concordance between provisional PTSD prevalence based on the diagnostic criteria for PTSD that are defined using DSM-IV, DSM-5, and proposed ICD-11. This supports the assumption of a set of PTSD core symptoms as suggested in the ICD-11 proposal, when at the same time a satisfactory concordance between ICD-11 proposal and DSM was given. The finding of increased provisional PTSD prevalence under ICD-11 proposal in contrast to ICD-10 can be of guidance for future epidemiological research on PTSD prevalence, especially concerning further investigations on the impact, appropriateness, and usefulness of the time criterion included in ICD-10 versus the consequences of its deletion as proposed for ICD-11.

## Abstract (es)

**Planteamiento**. Recientemente, se han introducido cambios en los criterios diagnósticos para el trastorno por estrés postraumático (TEPT) según el Manual Diagnóstico y Estadístico de los Trastornos Mentales (DSM) y la Clasificación Internacional de Enfermedades (CIE).Objetivos. Este estudio investigó el efecto de los cambios diagnósticos realizados del DSM-IV al DSM-5 y de la CIE-10 a la propuesta de la CIE-11. La concordancia de la prevalencia provisional del TEPT entre los criterios diagnósticos se examinó en una muestra de conveniencia de 100 miembros de las Fuerzas Armadas alemanas.

**Método**. Basándose en mediciones de cuestionarios, la prevalencia provisional del TEPT se evaluó de acuerdo con el DSM-IV, el DSM-5, la CIE-10 y los criterios propuestos por la CIE-11. Se evaluó estadísticamente la consistencia del estado diagnóstico en todos los sistemas de diagnóstico.

**Resultados**. La prevalencia provisional del TEPT fue la misma para el DSM-IV y el  DSM-5 (56%), y comparable en DSM-5 frente a la propuesta de la CIE-11 (48%), y el grado de acuerdo entre el DSM-IV y el DSM-5 y entre el DSM-5 y la propuesta de la CIE-11 fue alto (ambos p <0,001). La prevalencia provisional del TEPT aumentó significativamente en la propuesta de la CIE-11 en comparación con la CIE-10 (30%), debido principalmente a la supresión del criterio de tiempo. El grado de acuerdo entre la CIE-10 y la propuesta de la CIE-11 fue bajo (p = 0,014).

**Conclusión**. Este estudio proporciona evidencia preliminar de una concordancia satisfactoria entre la prevalencia provisional del TEPT basada en los criterios diagnósticos para el TEPT que se definen usando el DSM-IV, el DSM-5 y la propuesta de la CIE-11. Esto apoya que se asuman un conjunto de síntomas centrales del TEPT como se sugiere en la propuesta de la CIE-11, cuando al mismo tiempo se daba una concordancia satisfactoria entre la propuesta de la CIE-11 y el DSM. El hallazgo de un aumento de la prevalencia provisional de TEPT en la propuesta de la CIE-11 en contraste con la CIE-10 puede ser una guía para futuras investigaciones epidemiológicas sobre la prevalencia del TEPT, especialmente en relación con investigaciones adicionales sobre el impacto, la idoneidad y la utilidad del criterio de tiempo incluido en la CIE-10 frente a las consecuencias de su supresión, como se propone para la CIE-11.

## Abstract (zh)

背景：最近，《精神障碍的诊断和统计手册（DSM）》和《国际疾病分类（ICD）》对创伤后应激障碍（PTSD）的诊断标准发生了变化。

目标：本研究考察从DSM-IV到DSM-5，从 ICD-10到ICD-11提议的诊断改变。在一个方便取样的100名德国武装部队士兵样本中检验跨诊断标准间PTSD临时发生率的一致性。

方法：使用问卷测量，根据DSM-IV，DSM-5， ICD-10和提议的ICD-11标准计算PTSD临时发生率，并对跨诊断系统的诊断结果一致性进行统计评估。

结果：PTSD临时发生率在DSM-IV和DSM-5是一致的 (同为56%)，在DSM-5 中和提议的ICD-11（48%）结果相当。DSM-IV 和DSM-5之间，DSM-5和提议的ICD-11之间的统一性比较高 （都是p < .001）。 PTSD临时发生率从 ICD-10（30%）到提议的 ICD-11 提高了，主要是因为删除了时间标准。ICD-10和提议的ICD-11之间的统一性比较低（p = .014）。

结论：本研究使用DSM-IV，DSM-5和ICD-11提议中定义的PTSD诊断标准计算了PTSD临时发生率，提供了其具有令人满意的一致性的初步证据。这支持了ICD-11提议中对PTSD核心症状集的设想，同时提供了ICD-11提议和DSM之间的满意的一致性。ICD-11提议相比ICD-10提高了PTSD临时发生率，这个发现可以指导未来关于PTSD发病率的流行病学研究，尤其是未来对影响力、合适性的探讨，以及在ICD-10使用时间标准对比在ICD-11中删除时间标准的影响。

## 1. Introduction

In the last decade, there has been substantial criticism of the criteria for posttraumatic stress disorder (PTSD) in the 4th edition of the Diagnostic and Statistical Manual of Mental Disorders (DSM-IV; American Psychiatric Association, 2000) and the 10th revision of the International Classification of Mental and Behavioral Disorders (ICD-10; World Health Organization, 1993). First, concerns have been raised about the overlap of particular PTSD symptoms with symptoms of depression and anxiety (Maercker et al., 2013; Steel et al., 2009); second, a potential overuse of PTSD diagnoses in trauma-exposed populations has been discussed (Afana, 2012; Maercker et al., 2013; Steel et al., 2009); third, the trauma criterion has been criticized as not being adequately defined with respect to the selection of potentially traumatizing events (Breslau & Kessler, 2001; McNally, 2003; Rosen, 2004), as well as regarding the narrow interpretation of responses to trauma. PTSD can be associated with a wide range of reactions to trauma (Brewin, Andrews, & Rose, 2000; Kilpatrick et al., 1998) and can develop in the absence of responses of fear, helplessness, or horror (Adler, Wright, Bliese, Eckford, & Hoge, 2008; Breslau & Kessler, 2001). Thus, the already published 5^th^ edition of the DSM (DSM-5; American Psychiatric Association, 2013) as well as the proposal for the 11^th^ revision of the ICD (World Health Organization, 2012) introduced major changes to the diagnostic criteria for PTSD in adults that are described in detail below.

### 1.1 DSM-IV versus DSM-5

First, the DSM-5 (American Psychiatric Association, 2013) expanded the A1 criterion to ‘exposure to sexual violence’, and removed the A2 criterion due to insufficient clinical utility and limited predictive value (Friedman, 2013). This expands the context of PTSD to a disorder following a broader range of stressful events and including reactions associated with other states than fear or anxiety (Brewin et al., 2000; Friedman, Resick, Bryant, & Brewin, 2011). Second, the three symptom clusters known from DSM-IV were replaced by four symptom clusters by splitting the formerly known DSM-IV cluster C into two distinct categories (Cluster C: avoidance of stimuli; Cluster D: alterations in cognitions and mood) (Friedman, 2013; Friedman et al., 2011; Gentes et al., 2014). Moreover, the DSM-5 criteria D and E (formerly criterion D in DSM-IV) now comprise three additional symptoms that had not been included in DSM-IV, and two symptoms known from DSM-IV were rephrased for DSM-5. Thus, the number of qualifying and of necessarily endorsed symptoms differs between DSM-IV and DSM-5. According to DSM-IV, one re-experiencing, three avoidance, and two arousal- and reactivity-related symptoms need to be met out of 17 qualifying symptoms. Contrary, DSM-5 demands one re-experiencing, one avoidance, two cognition- and mood-related, and two arousal- and reactivity-related symptoms out of 20 qualifying symptoms. However, both versions require symptoms to be present for at least one month, and impairment in at least one area of functioning.

### 1.2 ICD-10 versus ICD-11 proposal

First, whereas the ICD-10 asks for one re-experiencing, one avoidance, and one feeling of continued threat symptom out of 17 qualifying symptoms, the ICD-11 proposal defines six qualifying symptoms, two on each of the three subscales only. This parsimonious conceptualization of PTSD aims at simplifying the assessment and at reducing over-diagnosing and false-positive comorbidities (Brewin, Lanius, Novac, Schnyder, & Galea, 2009; Cloitre, Garvert, Brewin, Bryant, & Maercker, 2013; Maercker et al., 2013; Stein, Seedat, Iversen, & Wessely, 2007), assuming that these symptoms represent characteristics that are salient to all PTSD cases (Brewin et al., 2009; Maercker et al., 2013). Besides, the ICD-11 proposal clarifies that impairment in one area of functioning and a duration of at least one month must be reported (Maercker et al., 2013). Moreover, the traumatic event does not need to cause immediate distress (Brewin et al., 2009; Maercker et al., 2013), and the symptom onset can be delayed more than six months post trauma (Andrews, Brewin, Philpott, & Stewart, 2007).

### 1.3 Epidemiological research

To date, literature evaluating the consistency between PTSD prevalence between the four diagnostic systems has yielded inconsistent results. The majority of publications comparing DSM-IV to DSM-5 report no differences (Carmassi et al., 2013; Elhai, Ford, Ruggiero, & Christopher Frueh, 2009; Elhai et al., 2012; Gentes et al., 2014; Kilpatrick et al., 2013; Miller et al., 2013; O’Donnell et al., 2014), with the exception of Forbes et al. (2011) who found lower PTSD prevalence under DSM-5. Of those who reported consistency (Carmassi et al., 2013; Elhai et al., 2009; Gentes et al., 2014; Kilpatrick et al., 2013), all reported satisfying high agreement between both versions of the DSM. Comparing the proposed ICD-11 to DSM-IV criteria, Stammel, Abbing, Heeke, and Knaevelsrud (2015) reported reduced PTSD prevalence according to the proposed ICD-11 criteria. In contrast, van Emmerik and Kamphuis (2011) as well as Morina, Emmerik, Andrews, and Brewin (2014) found no differences. To our knowledge, only two studies to date have systematically compared all four diagnostic systems, again yielding inconsistent results. Whereas Stein et al. (2014) found no differences in PTSD prevalence at all, O’Donnell et al. (2014) reported no differences between DSM-5 and DSM-IV, but lower PTSD prevalence under the proposed ICD-11 compared to DSM-IV, DSM-5, and ICD-10. Notably, although interpretation of prevalence differences between different diagnostic systems is limited when no consistency is reported, analyses of agreement between the diagnostic systems are provided only by some authors (Carmassi et al., 2013; Elhai et al., 2009; Gentes et al., 2014; Kilpatrick et al., 2013; Morina et al., 2014; Stammel et al., 2015).

War veterans and active soldiers represent a population at increased risk for PTSD since they are confronted with potentially traumatizing events almost daily. However, this population must show a high level of physical and mental fitness, emphasising the need for reliable and valid diagnostic systems and instruments and thus underlining the importance of investigating the concordance and appropriateness of the different diagnostic systems for this trauma population. However, we are aware of only a few studies that examined PTSD prevalence among veterans of war or active soldiers (Gentes et al., 2014; Miller et al., 2013; Morina et al., 2014; Wisco et al., 2016). Although, Gentes et al. (2014) and Miller et al. (2013) report comparable PTSD prevalence between DSM-IV and DSM-5, and Morina et al. (2014) found comparable PTSD prevalence between the ICD-11 proposal and DSM-IV, Wisco et al. (2016) report significantly reduced PTSD prevalence under the ICD-11 proposal compared to DSM-5 as well as compared to ICD-10, indicating an unsatisfactory concordance between these systems. However, no simultaneous comparison of all these diagnostic systems, i.e. the ICD-11 proposal, ICD-10, DSM-IV, and DSM-5, is available.

The main purpose of this study was to expand the empirical evidence on concordance of PTSD prevalence between the diagnostic systems DSM-IV, DSM-5, ICD-10, and the ICD-11 proposal. We focused on the population of war veterans and active soldiers by recruiting treatment-seeking members of the German Armed Forces (GAF) with reported lifetime traumatization. Of special concern for this study was the concordance when self-rated questionnaires were scored following the diagnostic rules of DSM-IV, DSM-5, ICD-10, and proposed ICD-11 criteria for PTSD. It is of note that most earlier studies in this area used clinician-administered interviews to check for a positive diagnosis of PTSD (Elhai et al., 2009; Forbes et al., 2011; Gentes et al., 2014; Morina et al., 2014; O’Donnell et al., 2014; Stein et al., 2014; van Emmerik & Kamphuis, 2011; Wisco et al., 2016). While this is without doubt the gold standard for clinical research, clinical practice often heavily relies on self-administered instruments, underlining the importance of investigating the consistency when self-rating instruments for PTSD are provided. Based on research findings, we expected that the PTSD prevalence would be the same using DSM-IV versus DSM-5 criteria (Carmassi et al., 2013; Elhai et al., 2012; Gentes et al., 2014; Kilpatrick et al., 2013; Miller et al., 2013; O’Donnell et al., 2014; Stein et al., 2014), but would be reduced under the ICD-11 proposal as compared to ICD-10 and DSM-5 (O’Donnell et al., 2014; Wisco et al., 2016).

## 2. Method

### 2.1. Participants and procedure

Data were collected in a convenience sample of 100 treatment-seeking members of the GAF who had returned from deployment, were over the age of 18, reported a history of lifetime traumatization, and were fluent in German. Participants were recruited and assessed between June 2014 and February 2015 in collaboration with the inpatient and outpatient clinics of the GAF hospital in Berlin. Of the patients invited to the study, 57% agreed to and participated in the study. Participants consented to participate after they had been informed about the study’s content, data confidentiality, and anonymity. Data were collected by utilizing paper-and-pencil questionnaires. Participants were told that they would receive a number of questionnaires that deal with different aspects of physical and mental health. Further, they were instructed that although some of the questions throughout the questionnaires may seem to be very similar, they should not feel confused by this, and that they must answer each item. The questionnaires of interest for the present study were part of a larger survey, so that the presentation of the questionnaires of interest was not back-to-back but interleaved by other inventories, reducing the risk of order effects. First, after filling in a short questionnaire on demographic information, participants filled in the German version of the Life Events Checklist for DSM-5 (LEC-5; Weathers et al., 2013a; German version: Appendix), and the German version of the Posttraumatic Stress Disorder Checklist for DSM-5 (PCL-5; Weathers et al., 2013; German version: Ehring, Knaevelsrud, Krüger, & Schäfer, 2014). Afterwards, six distinct inventories of 219 items in total were given to the participants. Finally, the participants received the German version of the Posttraumatic Stress Diagnostic Scale (PDS; Foa, 1995; German version: PDS-D; Ehlers, Steil, Winter, & Foa, 1996). The study was approved by the Review Board of the University of Muenster.

Participants were on average 35.22 years old (*SD = *8.84) and predominantly male (86%). Most participants lived together with a partner (60%) or in a single-household (24%). Subjects reported being in a relationship (32%), married (37%), single (21%), or divorced (10%). Two-thirds were employed full-time (66%), whereas the remaining worked part-time (6%), were unemployed (5%), retired (3%), or studying/on parental leave/unfit for work (18%); two participants gave no information.

### 2.2. Measures

#### 2.2.1. PTSD symptoms

The German version of the Posttraumatic Stress Diagnostic Scale (PDS; Foa, 1995; German version: PDS-D; Ehlers et al., 1996) was used to assess PTSD symptoms and provisional PTSD diagnostic status referring to DSM-IV and ICD-10. Section 3 of the PDS-D assesses PTSD symptoms during the past month based on 17 items on a 4-point scale (0 = ‘never/only once during the past month’; 3 = ‘5 times per week or more/nearly always’). Participants’ ratings of 1 (‘once a week or less/once in a while’) or higher indicated that a symptom was endorsed. Section 4 checks for impairment in at least one area of functioning. Participants were instructed to complete the PDS-D based on a ‘worst event that still troubles them the most today’. The PDS-D is one of the most commonly used and well validated instruments to assess PTSD, as supported by Griesel, Wessa, and Flor (2006) who reported satisfactory psychometric properties and high internal consistency (.88 < α < .94 for symptom clusters and total scale). In this study, Cronbach’s alpha was satisfactory (total scale α = .95; intrusion α = .94; avoidance α = .89; hyperarousal α = .86).

The German version of the Posttraumatic Stress Disorder Checklist for DSM-5 (PCL-5; Weathers et al., 2013; German version: Ehring et al., 2014) was used to assess PTSD symptoms and provisional PTSD diagnostic status following DSM-5 and the ICD-11 proposal. Twenty items assess PTSD symptoms on a 5-point scale (0 = ‘not at all’; 4 = ‘extremely’), whereby all questions refer to the past month. Participants’ ratings of 2 (‘moderately’) or higher indicated that a symptom was endorsed. Participants were instructed to complete the PCL-5 based on a ‘worst event that still troubles them the most today’. The PCL-5 was developed based on the DSM-5 criteria, and preliminary psychometric evaluations revealed high internal consistency (α = .94), good test-retest reliability (.56 < *r* < .82), and high discriminability and convergence (Blevins, Weathers, Davis, Witte, & Domino, 2015; Krüger-Gottschalk et al., 2016). In the current study, internal consistency was satisfactory (total scale α = .97; intrusion α = .93; avoidance α = .88; cognitions and mood α = .91; hyperarousal α = .89).

#### 2.2.2. Trauma exposure

Traumatic events were measured using the trauma list of the PDS-D providing 11 traumatic events as well as by providing the German version of the Life Events Checklist for DSM-5 (LEC-5; Weathers et al., 2013a; German version: Appendix) providing 17 traumatic events. In both instruments, participants were asked to name one worst event that troubles them the most today.

#### 2.2.3. Provisional diagnostic status based on DSM-IV versus DSM-5

For a provisional diagnosis based on DSM-IV, participants had to endorse one re-experiencing, three avoidance, and two hyperarousal symptoms out of 17 qualifying symptoms for the past month, with symptom ratings of 1 or higher on the PDS-D. They had to report feelings of fear, helplessness, or horror during trauma exposure, as well as current impairment in at least one area of functioning. For a provisional diagnosis based on DSM-5, participants needed to meet one re-experiencing, one avoidance, two alterations in cognition and mood, and two alterations in arousal and reactivity symptoms out of 20 qualifying symptoms for the past month, with symptom ratings of 2 or higher on the PCL-5. Current impairment in at least one area of daily functioning was required.

#### 2.2.4. Provisional diagnostic status based on ICD-10 versus the ICD-11 proposal

For a provisional diagnosis based on ICD-10, participants had to endorse one re-experiencing, one avoidance, and one hyperarousal symptom out of 17 qualifying symptoms, with symptom ratings of 1 or higher on the PDS-D. Participants had to report distress during trauma exposure, and symptom onset within six months post trauma. For receiving a provisional diagnosis based on the ICD-11 proposal, we followed the suggestions put forward by Brewin et al. (2009) and Maercker et al. (2013): Participants needed to fulfil one re-experiencing, one avoidance, and one sense of threat symptom out of six qualifying symptoms, with symptom rating of 2 or higher on the PCL-5. Symptoms had to be present for at least one month, and current impairment in at least one area of functioning was required.

### 2.3. Data analysis

Analyses were conducted using SPSS 22.0 (IBM Corporation, 2013). As there was only a small amount of data (0.2%) that was missing at random, the performance of an expectation-maximization algorithm was justified to impute a single new data set without missing data. We calculated the proportions of participants meeting the diagnostic criteria for justifying a provisional PTSD diagnosis under DSM-IV, DSM-5, ICD-10, and the ICD-11 proposal. We then calculated the proportion of participants changing (i.e. gaining or losing) or maintaining the provisional diagnostic status when the transition from DSM-IV to DSM-5, from ICD-10 to ICD-11, and from DMS-5 to the ICD-11 proposal was applied. Two-tailed binomial-approximation tests for proportions were applied for PTSD prevalence between the different diagnostic systems, and Cohen´s kappa was calculated for concordance between the different diagnostic systems. Significance was set at *p *< .05 for all analyses.

## 3. Results

### 3.1. Trauma exposure

On average, 4.14 (*SD* = 1.61) traumatic events in the PDS-D and 9.02 (*SD* = 3.54) events in the LEC-5 were reported. The most frequently reported events were exposure to serious accident/fire/explosion (84%), deployment to or battle action in an area of war (84%), and severe human suffering (78%), all of which took place in a military context, and they were at the same time those events that still troubled them the most today. On average, 5.93 (*SD *= 5.47) years had passed since the traumatic event. Whereas 53.1% of participants reported that they experienced symptoms such as irritability, sleep disturbances, intrusive thoughts, or flashbacks within the first six months post trauma, the remainder reported a late symptom onset.

### 3.2. Provisional diagnosis based on DSM-IV versus DSM-5

The prevalence of provisional PTSD was the same under DSM-IV and DSM-5 (Table 1). Eleven participants gained the provisional diagnosis when the transition from DSM-IV to DSM-5 was made, whereas another 11 participants lost it. The difference was not significant (*p *= .54), and level of agreement was satisfactory (78%, *κ *= .55, *p* < .001). Table 1 illustrate the concordance between both systems. Participants who lost the diagnosis did not meet the required DSM-5 symptoms of negative alterations in cognitions and mood (*N = *9, 81.8%), alterations in arousal and reactivity (*N = *7, 63.6%), avoidance (*N = *6, 54.5%), or re-experiencing (*N *= 2, 18.2%). Two (18.2%) participants gained the diagnosis under DSM-5 due to the deletion of the A2 criterion, the remaining changes were attributable to differences in symptom requirements between both versions. No differences between participants that received the provisional diagnosis under DSM-IV but not under DSM-5 and vice versa were found regarding age, gender, time since trauma, number of traumatic events, and mean PTSD symptom severity (.152 ≤ *p* ≤ .949).

**Table 1.** Prevalence of provisional PTSD diagnosis based on DSM-IV and DSM-5, *N* = 100.

Prevalence (*N*, %) of provisional diagnosis based on DSM-IV ^a^		Prevalence (*N*, %) of provisional diagnosis based on DSM-5 ^b^	
		Diagnosis given	Diagnosis not given
		56 (56.0%)	44 (44.0%)
Diagnosis given	56 (56.0%)	45 (80.4%)	11 (19.6%)
Diagnosis not given	44 (44.0%)	11 (25.0%)	33 (75.0%)

^a^ Proportions based on PDS-D; ^b^ Proportions based on PCL-5.

### 3.3. Provisional diagnoses based on ICD-10 versus ICD-11 proposal

Significantly more participants met the criteria for a provisional PTSD diagnosis under the ICD-11 proposal (48%) than under ICD-10 (30%) (*p *< .001). As depicted in Table 2, 28 participants gained a provisional diagnosis when moving from ICD-10 to the ICD-11 proposal, whereas 10 lost it. Agreement was low (62%, *κ* = .228, *p *= .014). Table 2 illustrates the concordance between both diagnostic systems. Participants who lost their provisional diagnosis did not meet the proposed ICD-11 criterion of re-experiencing (*N = *7, 70%), alterations in sense of threat (*N = *4, 40%), or avoidance (*N = *3, 30%). In contrast, 24 (85.7%) participants gained the provisional diagnosis due to the deletion of the time criterion, and two (7.1%) reported reactions to trauma that did not involve high distress. The remaining changes were attributable to differences in symptom requirements between both versions. No differences between participants that received the provisional diagnosis under ICD-10 but not under the ICD-11 proposal and vice versa were found regarding age, gender, time since trauma, number of traumatic events, and mean PTSD symptom severity (.233 ≤ *p* ≤ .951).

**Table 2.** Prevalence of provisional PTSD diagnosis based on ICD-10 and ICD-11 proposal, *N* = 100.

Prevalence (*N*, %) of provisional diagnosis based on ICD-10 ^a^		Prevalence (*N*, %) of provisional diagnosis based on ICD-11 proposal ^b^	
		Diagnosis given	Diagnosis not given
		48 (48%)	52 (52%)
Diagnosis given	30 (30%)	20 (66.7%)	10 (33.3%)
Diagnosis not given	70 (70%)	28 (40.0%)	42 (60.0%)

^a^ Proportions based on PDS-D; ^b^ Proportions based on PCL-5.

### 3.4. Provisional diagnostic status based on DSM-5 versus the ICD-11 proposal

The difference in provisional PTSD prevalence under DSM-5 (56%) versus ICD-11 proposal (48%) was not significant (*p *= .066). Table 3 illustrates the concordance between both diagnostic systems. As can be seen, nine participants lost their diagnostic status under the ICD-11 proposal, whereas only one gained it. Eight (88.9%) did not meet the criterion for re-experiencing and two (22.2%) did not meet the criterion for alterations in arousal and sense of threat under the ICD-11 proposal. However, agreement was satisfactory (90%, *κ *= .801, *p *< .001). No differences between participants that received the provisional diagnosis under the ICD-11 proposal but not under DSM-5 and vice versa were found regarding age, gender, time since trauma, number of traumatic events, and mean PTSD symptom severity (.182 ≤ *p* ≤ .922).

**Table 3.** Prevalence of provisional PTSD diagnosis based on ICD-11 proposal and DSM-5, *N* = 100.

Prevalence (*N*, %) of provisional diagnosis based on DSM-5 ^a^		Prevalence (*N*, %) of provisional diagnosis based on ICD-11 proposal ^a^	
		Diagnosis given	Diagnosis not given
		48 (48%)	52 (52%)
Diagnosis given	56 (56%)	47 (83.9%)	9 (16.1%)
Diagnosis not given	44 (44%)	1 (2.3%)	43 (97.7%)

^a^ Proportions based on PCL-5.

## 4. Discussion

In line with our hypothesis and consistent with previous findings (Carmassi et al., 2013; Elhai et al., 2009, 2012; Gentes et al., 2014; Kilpatrick et al., 2013; Miller et al., 2013; O’Donnell et al., 2014; Stein et al., 2014), no change in provisional PTSD prevalence was identified when the criteria shifted from DSM-IV to DSM-5. Although, DSM-IV and DSM-5 include a different number of qualifying symptoms, group these symptoms into specific clusters, and thus implicitly demand specific symptom characteristics to be present in a minimum number and specific combination, possibly leading to the identification of somewhat different patient populations in the present study, the agreement between both systems was satisfactory. Although, this may raise the question of the necessity and appropriateness of the changes made to DSM, earlier research that dealt with latent factor structures supported the four-factor approach that is now implemented in the DSM-5 (Forbes et al., 2011; Gentes et al., 2014; Miller et al., 2013). However, in the current study the deletion of the A2 criterion contributed to a diagnostic change for some participants that have met all required symptoms but did not report fear, horror, or helplessness during traumatization. This finding supports earlier research that reveals that a proportion of trauma survivors with clinically significant PTSD symptoms report a range of peri-traumatic reactions different from fear or helplessness, indicating a limited prognostic value of the A2 criterion for the development of PTSD, and suggesting an extension of the range of possible peri-traumatic reactions (Brewin et al., 2000; Friedman, 2013; Friedman et al., 2011).

In contrast to our assumption and to earlier research (O’Donnell et al., 2014; Stein et al., 2014; Wisco et al., 2016), the provisional PTSD prevalence was increased under the ICD-11 proposal compared to ICD-10. However, the increase was mainly due to the deletion of the time criterion, accounting for a tendency of late symptom onset in the present sample. This finding provides further preliminary support for the deletion of the time criterion and supports a systematic review that reports on delayed PTSD onset, particularly among individuals exposed to combat or war (Andrews et al., 2007). One might think of underlying mechanisms that may facilitate a late symptom onset, especially among populations of military personnel that are presented with long lasting and repeated traumatization. Possibly, during or immediately after this ongoing and repeated traumatization these individuals may be able to compensate for the psychological stress, keeping their physical and mental fitness as high as possible, and thus placing them at a higher chance of survival during these tough times. However, their psychological resilience may be significantly reduced on a sustained basis, making them even more vulnerable to stressors and crises that in turn may have the potential to activate PTSD later in life, long after the traumatic event or period has ended. However, this assumption needs further evaluation and future research dealing with the mechanism of late-onset PTSD in diverse populations of trauma survivors.

Although the proposed ICD-11 criteria include only six qualifying symptoms, while the DSM-5 includes 20, the results of the current study indicate an overall satisfactory agreement between both systems. This finding of the current study contrasts with Wisco et al. (2016) who found significantly reduced PTSD prevalence under the ICD-11 proposal compared to DSM-5. This significant reduction of qualifying symptoms under the ICD-11 proposal when at the same time the concordance between both systems is still satisfactory gives preliminary reason to assume that the parsimonious collection of PTSD symptoms under ICD-11 (Brewin et al., 2009; Maercker et al., 2013) may be appropriate and reliable. This is in line with a review providing evidence that PTSD screening instruments with fewer items can perform as well as or even better than longer and more complex measures (Brewin, 2005).

However, future research is needed to further verify the adequacy and sufficiency of the six core symptoms that are chosen for the ICD-11 proposal. Furthermore, since both diagnostic criteria seem to fit equally well to the present sample, the question arises whether there is a ‘latent’ PTSD towards which the different diagnostic systems are iteratively approaching (Kendler, Zachar, & Craver, 2011). Kendler et al. (2011) argue that psychological processes and structures may be underlying the phenotypes of psychiatric disorders demanding some degree of abstraction that may be solved by diagnostic systems. Further research is needed to shed a deeper light on the question whether this abstraction may be portrayed in the most concise way in the ICD-11 proposal, as suggested by earlier research (Brewin et al., 2009; Maercker et al., 2013).

The current study expands the field of research that deals with populations of war veterans or active soldiers (Gentes et al., 2014; Miller et al., 2013; Morina et al., 2014). Whereas the findings of the current study support earlier findings of comparable PTSD prevalence under DSM-IV and DSM-5 (Gentes et al., 2014; Miller et al., 2013), the study´s findings concerning the transition from DSM-5 to the ICD-11 proposal as well as from ICD-10 to the ICD-11 proposal are innovative and add knowledge to research and to the literature. Moreover, the current study expands the field of research that compares PTSD prevalence among all four diagnostic systems (O’Donnell et al., 2014; Stein et al., 2014). Although the current study contributes to the inconsistency of research findings that is reported to date, its results preliminarily support the diagnostic changes made to DSM and to ICD. However, future research is needed to strengthen our findings.

### 4.1. Limitations

Several limitations of the current study need to be mentioned. First, PTSD diagnostic status was based on self-report questionnaires only and therefore can provide estimations of probable PTSD prevalence only. Although verification of the provisional diagnostic status by application of structured clinical interviews such as the Clinician Administered PTSD Scale for DSM-5 (CAPS-5; Weathers et al., 2013b) is generally regarded as the gold standard, research has shown good agreement between PTSD diagnoses based on self-report questionnaires and on clinical interviews (e.g. Ehring, Kleim, Clark, Foa, & Ehlers, 2007). Self-report ratings represent an important component in clinical practice and research today, thus underlining the high relevance of the current study to specifically evaluate the concordance of provisional PTSD diagnostic status that is based on well-established self-report inventories.

Second, in the current study two different diagnostic instruments were utilized, namely the PDS-D for DSM-IV and ICD-10, and the PCL-5 for DSM-5 and the ICD-11 proposal. We cannot rule out that differences between the provisional prevalence of PTSD reported in the present study may be partly due to differences between the diagnostic instruments, i.e. both systems require different symptom severity ratings to count a symptom as being endorsed, which makes it hard to tell whether the participants understood ‘once a week or less/once in a while’ in the same way they interpreted ‘moderately’. However, the application of the PDS-D and the PCL-5 was justified since the PDS-D is one of the most commonly used and well validated instruments to assess PTSD referring to ICD-10 and DSM-IV criteria (Griesel et al., 2006), and the PCL-5 was developed based on the DSM-5 criteria. However, at the time of planning the current study, no instrument was yet available to assess the proposed ICD-11 criteria (Brewin et al., 2009; Maercker et al., 2013). We are aware that in the meantime an instrument assessing the proposed ICD-11 criteria was developed (Cloitre, Roberts, Bisson, & Brewin, 2015) that has been used in recent research (Dokkedahl, Oboke, Ovuga, & Elklit, 2015). However, this instrument has not been well enough established and validated up to now.

Finally, the sample was a comparably small convenience sample and one might argue that the study was not sufficiently powered. However, the current study aims specifically at the population of GAF, to add knowledge to the field of research that deals with military personnel as a specific population that is at increased risk for PTSD due to ongoing, repeated, and work-related trauma exposure (Gentes et al., 2014; Miller et al., 2013; Morina et al., 2014). With respect to the scarce literature that deals with PTSD prevalence concordance between DSM-IV, DSM-5, ICD-10, and ICD-11 proposal in military personnel to date, the current study should be considered as an exploratory approach providing some guidance for future investigations to corroborate our findings.

### 4.2. Conclusion

The current study provides preliminary evidence for the impact that the changes of the DSM and the ICD diagnostic criteria for PTSD can have on the diagnostic status in a population of GAF that is exposed to military-related traumatic experiences, which is of relevance for future investigations on measuring and studying PTSD. On the one hand, promising results are provided regarding the concordance between DSM-5 and proposed ICD-11 criteria, and between DSM-IV and DSM-5, as well as concerning the appropriateness of changes made to DSM and ICD in general. On the other hand, the concordance between DSM-IV and DSM-5 as well as between DSM-5 and proposed ICD-11 raises the question of a ‘latent’ PTSD structure that may be underlying the well-known broad diagnostic instruments and that may be found in a more parsimonious concept of PTSD, that may be approached by the proposed ICD-11 criteria.

## Appendix

**LEC-5**

Nachfolgend sind eine Anzahl schwieriger oder belastender Dinge aufgelistet, die Menschen manchmal zustoßen. Kreuzen Sie für jedes Ereignis eines oder mehrere Felder auf der rechten Seite an, um anzugeben, dass (a) es Ihnen persönlich zugestoßen ist; (b) Sie Zeuge davon waren, als es jemand anderem zugestoßen ist; (c) Sie davon erfahren haben, dass es einem nahen Angehörigen oder engen Freund zugestoßen ist; (d) Sie damit im Rahmen Ihres Berufes konfrontiert wurden (z.B. Rettungssanitäter, Polizist, Soldat oder anderer Ersthelfer); (e) Sie unsicher sind, ob es zutrifft; oder (f) es auf Sie nicht zutrifft.

Bitte achten Sie darauf, Ihr gesamtes Leben zu berücksichtigen (Kindheit/Jugend und Erwachsenenalter), wenn Sie die Liste der Ereignisse durchgehen.

**Table:**

*Ereignis*	*mir persönlich zugestoßen*	*Zeuge davon gewesen*	*davon erfahren*	*im Rahmen meines Berufs*	*unsicher*	*trifft nicht zu*
**1. Naturkatastrophe (z.B. Überschwemmung, Orkan, Tornado, Erdbeben)**						
**2. Feuer oder Explosion**						
**3. Verkehrsunfall (z.B. Autounfall, Schiffsunglück, Zugunglück, Flugzeugabsturz)**						
**4. Schwerer Unfall bei der Arbeit, zuhause oder während einer Freizeitaktivität**						
**5. Einem Schadstoff ausgesetzt sein (z.B. gefährliche Chemikalien, Strahlung)**						
**6. Gewalttätiger Angriff (z.B. überfallen, geschlagen, getreten oder zusammengeschlagen werden)**						
**7. Angriff mit einer Waffe (z.B. verletzt oder bedroht werden mit einer Schusswaffe, einem Messer oder einer Bombe)**						
**8. Sexueller Übergriff (Vergewaltigung, versuchte Vergewaltigung, zu irgendeiner Art von sexueller Handlung durch Gewalt oder Androhung von Gewalt gezwungen werden)**						
**9. Andere unerwünschte oder unangenehme sexuelle Erfahrung**						
**10. Kampfhandlungen oder Aufenthalt in einem Kriegsgebiet (beim Militär oder als Zivilist)**						
**11. Gefangenschaft (z.B. gekidnappt, entführt, als Geisel genommen werden, Kriegsgefangener)**						
**12. Lebensbedrohliche Erkrankung oder Verletzung**						
**13. Schweres menschliches Leid**						
**14. Plötzlicher gewalttätiger Tod (z.B. Mord, Suizid)**						
**Plötzlicher Unfalltod**						
**15. Schwere Verletzung, Schaden oder Tod, die/den Sie jemand anderem zugefügt haben**						
**16. Irgendein anderes sehr belastendes Ereignis oder Erlebnis**						

**TEIL 2:**

**A. Falls Sie irgendetwas bei Nr. 17 in TEIL 1 angekreuzt haben, benennen Sie kurz das Ereignis, an das Sie gedacht haben:**

**_________________________________________________________________________________________________________________________**

**B. Falls Sie mehr als eines der in TEIL 1 genannten Ereignisse erlebt haben, denken Sie bitte an das Ereignis, das Sie als das *schlimmste Ereignis* betrachten; das bedeutet für diesen Fragebogen das Ereignis, das Sie zurzeit am meisten belastet. Falls Sie nur eines der in TEIL 1 genannten Ereignisse erlebt haben, nehmen Sie dieses als das schlimmste Ereignis. Bitte beantworten Sie die folgenden Fragen in Bezug auf das schlimmste Ereignis (*kreuzen Sie alle Auswahlmöglichkeiten an, die zutreffen):***

**1. Beschreiben Sie kurz das schlimmste Ereignis (z.B. was passierte, wer beteiligt war, usw.)**

__________________________________________________________________________________________________________________________

__________________________________________________________________________________________________________________________

**2. Wie lange ist es her? ____________________ (*Bitte schätzen, falls Sie sich nicht sicher sind*)**

**3. Auf welche Weise haben Sie es erlebt?**

*__ Es ist mir selbst passiert.*

*__ Ich habe es beobachtet*

*__ Ich habe erfahren, dass es einem nahen Angehörigen oder engen Freund passiert ist*

*__ Ich wurde im Rahmen meines Berufes wiederholt mit Details des Ereignisses konfrontiert (z.B. Rettungssanitäter, Polizist, Soldat oder anderer Ersthelfer)*

*__ Sonstiges, bitte beschreiben: _________*

**4. War jemand in Lebensgefahr?**

*__ Ja, ich*

*__ Ja, jemand anderes*

*__ Nein*

**5. Wurde jemand schwer verletzt oder getötet?**

*__ Ja, ich wurde schwer verletzt*

*__ Ja, jemand anderes wurde schwer verletzt oder getötet*

*__ Nein*

**6. Beinhaltete es sexuelle Gewalt?** *___ Ja ___ Nein*

**7. Falls das Ereignis den Tod eines nahen Angehörigen oder engen Freundes beinhaltete, war das die Folge eines Unfalls oder von Gewalt, oder war es die Folge natürlicher Umstände?**

*__ Unfall oder Gewalt*

*__ Natürliche Umstände*

*__ Nicht zutreffend (Das Ereignis beinhaltete nicht den Tod eines nahen Angehörigen oder Freundes)*

**8. Wie häufig haben Sie insgesamt ein ähnliches Ereignis erlebt, das genauso belastend oder fast genauso belastend war wie das schlimmste Ereignis?**

*__ Nur einmal*

*__ Mehr als einmal (Bitte nennen oder schätzen Sie die Anzahl, wie häufig Sie dieses Erlebnis hatten: ____)*

**TEIL 3: Nachfolgend sind Probleme aufgelistet, die Menschen manchmal als Reaktion auf ein sehr belastendes Erlebnis haben. Bitte lesen Sie jedes Problem sorgfältig, denken Sie dabei an Ihr schlimmstes Ereignis, und markieren Sie dann eine der Zahlen auf der rechten Seite um anzugeben, wie stark Sie im letzten Monat durch dieses Problem belastet waren**.

**Table:**

*Im letzten Monat, wie sehr waren Sie belastet durch:*	überhaupt nicht	ein wenig	ziemlich	stark	sehr stark
**1. Wiederholte, beunruhigende und ungewollte Erinnerungen an das belastende Erlebnis?**	**0**	**1**	**2**	**3**	**4**
**2. Wiederholte, beunruhigende Träume von dem belastenden Erlebnis?**	**0**	**1**	**2**	**3**	**4**
**3. Sich plötzlich fühlen oder sich verhalten, als ob das belastende Erlebnis tatsächlich wieder stattfinden würde (*als ob Sie tatsächlich wieder dort wären und es wiedererleben würden)*?**	**0**	**1**	**2**	**3**	**4**
**4. Sich emotional sehr belastet fühlen, wenn Sie etwas an das Erlebnis erinnert hat?**	**0**	**1**	**2**	**3**	**4**
**5. Starke körperliche Reaktionen, wenn Sie etwas an das belastende Erlebnis erinnert hat *(z.B. Herzklopfen, Schwierigkeiten beim Atmen, schwitzen*)**	**0**	**1**	**2**	**3**	**4**
**6. Vermeidung von Erinnerungen, Gedanken oder Gefühlen in Bezug auf das belastende Erlebnis?**	**0**	**1**	**2**	**3**	**4**
**7. Vermeidung äußerer Auslöser für Erinnerungen an das belastende Erlebnis *(z.B. Personen, Plätze, Gespräche, Aktivitäten, Gegenstände oder Situationen*)?**	**0**	**1**	**2**	**3**	**4**
**8. Schwierigkeiten, sich an wichtige Teile des belastenden Erlebnisses zu erinnern?**	**0**	**1**	**2**	**3**	**4**
**9. Starke negative Überzeugungen über sich selbst, andere Menschen oder die Welt *(z.B. Gedanken wie: Ich bin schlecht, mit mir stimmt ernsthaft etwas nicht, man kann niemandem vertrauen, die Welt ist absolut gefährlich)*?**	**0**	**1**	**2**	**3**	**4**
**10. Sich selbst oder jemand anderem Vorwürfe machen in Bezug auf das belastende Erlebnis oder was danach passiert ist?**	**0**	**1**	**2**	**3**	**4**
**11. Starke negative Gefühle, wie zum Beispiel Angst, Schrecken, Ärger, Schuld oder Scham?**	**0**	**1**	**2**	**3**	**4**
**12. Verlust von Interesse an Aktivitäten, die Ihnen früher Spaß gemacht haben?**	**0**	**1**	**2**	**3**	**4**
**13. Sich von anderen Menschen entfernt oder wie abgeschnitten fühlen?**	**0**	**1**	**2**	**3**	**4**
**14. Schwierigkeiten, positive Gefühle zu erleben *(z.B. keine Freude empfinden können oder keine liebevollen Gefühle haben können gegenüber Menschen, die Ihnen nahestehen)*?**	**0**	**1**	**2**	**3**	**4**
**15. Reizbares Verhalten, Wutausbrüche oder aggressives Verhalten?**	**0**	**1**	**2**	**3**	**4**
**16. Zu viele Risiken eingehen oder Dinge tun, die Ihnen Schaden zufügen könnten?**	**0**	**1**	**2**	**3**	**4**
**17. In erhöhter Alarmbereitschaft, wachsam oder auf der Hut sein?**	**0**	**1**	**2**	**3**	**4**
**18. Sich nervös oder schreckhaft fühlen?**	**0**	**1**	**2**	**3**	**4**
**19. Konzentrationsschwierigkeiten?**	**0**	**1**	**2**	**3**	**4**
**20. Schwierigkeiten, ein- oder durchzuschlafen?**	**0**	**1**	**2**	**3**	**4**
